# Competing Magnetic Interactions and Field-Induced Metamagnetic Transition in Highly Crystalline Phase-Tunable Iron Oxide Nanorods

**DOI:** 10.3390/nano13081340

**Published:** 2023-04-12

**Authors:** Supun B. Attanayake, Amit Chanda, Thomas Hulse, Raja Das, Manh-Huong Phan, Hariharan Srikanth

**Affiliations:** 1 Department of Physics, University of South Florida, Tampa, FL 33620, USA; attanayake@usf.edu (S.B.A.); sharihar@usf.edu (H.S.); 2 Department of Physics & Astronomy, University of Louisville, Louisville, KY 40208, USA; thomas.hulse@louisville.edu; 3 SEAM Research Centre, South East Technological University, X91 K0EK Waterford, Ireland; raja.das@wit.ie

**Keywords:** magnetic nanorods, biphase iron oxide, Verwey transition, Morin transition, annealing, magnetic hyperthermia, spintronics, metamagnetic transition

## Abstract

The inherent existence of multi phases in iron oxide nanostructures highlights the significance of them being investigated deliberately to understand and possibly control the phases. Here, the effects of annealing at 250 °C with a variable duration on the bulk magnetic and structural properties of high aspect ratio biphase iron oxide nanorods with ferrimagnetic Fe_3_O_4_ and antiferromagnetic α-Fe_2_O_3_ are explored. Increasing annealing time under a free flow of oxygen enhanced the α-Fe_2_O_3_ volume fraction and improved the crystallinity of the Fe_3_O_4_ phase, identified in changes in the magnetization as a function of annealing time. A critical annealing time of approximately 3 h maximized the presence of both phases, as observed via an enhancement in the magnetization and an interfacial pinning effect. This is attributed to disordered spins separating the magnetically distinct phases which tend to align with the application of a magnetic field at high temperatures. The increased antiferromagnetic phase can be distinguished due to the field-induced metamagnetic transitions observed in structures annealed for more than 3 h and was especially prominent in the 9 h annealed sample. Our controlled study in determining the changes in volume fractions with annealing time will enable precise control over phase tunability in iron oxide nanorods, allowing custom-made phase volume fractions in different applications ranging from spintronics to biomedical applications.

## 1. Introduction

Since the groundbreaking revelation of the concept in the 1960s by Richard Feynman, nanoparticles have made significant strides in many fields, especially magnetic nanoparticles have attracted significant attention for their potential applications in drug delivery, cancer treatment, and hyperthermia [1,2,3,4,5,6,7]. The scope of these fields has recently expanded into spintronic devices as well, which cover a wide variety of applications such as storing, processing, and transmitting data as well as in the energy sector showing potential applications as effective catalysts in fuel cells [8,9,10,11,12,13,14]. The iron oxide nanoparticles which are discussed here are well-known in all the above-mentioned fields as they are non-toxic, stable, able to functionalize, and possess a high surface–volume ratio [15,16,17,18]. Additionally, different phases of iron oxide such as α-Fe_2_O_3_, γ-Fe_2_O_3_, Fe_3_O_4_, etc. possess distinct magnetic ordering. These phases enable iron oxide nanoparticles and thin films to be customized to variable applications [19,20,21,22,23,24,25]. 

High aspect ratio nanorods synthesized by Das et al. [25]. illustrated exotic magnetic and inductive heating properties mainly due to the high surface–volume ratio and anisotropy. The epitaxial growth of these highly oriented Fe_3_O_4_ nanorods on SrTiO_3_ substrates has created a novel heterostructure with enhanced perpendicular magnetic anisotropy, which is desirable for magnetic data storage and spintronic devices [12]. Functionalizing the structure not only enhances its basic capabilities but opens a wide range of additional applications. For example, usage as a contrast agent in magnetic resonance imaging, as a gas sensor, or as an anode in battery cells [26,27,28,29]. To further extend the functionalization of iron oxide nanorods, phase, and structural tunability are necessary. A rigorous examination of iron oxide nanorods via magnetometry enabled Attanayake et al. [19]. to determine the presence of biphase which is undetectable when using orthodox characterization methods such as X-Ray Diffractometry (XRD). These results proposed the opportunity to study the phase tunability and coexistence of antiferromagnetic (AFM) α-Fe_2_O_3_ and ferrimagnetic Fe_3_O_4_. α-Fe_2_O_3_ is thermodynamically more stable and its changes with varying annealing temperatures in the presence of a free flow of oxygen were initially checked to determine an ideal formation temperature, which preserves the magnetic qualities of Fe_3_O_4_ but leverages the characteristics of α-Fe_2_O_3_ [29,30,31]. This temperature was later determined to be 250 °C and has been utilized in all the annealing runs in the current experimental setup which was annealed for varying durations in the presence of a free flow of oxygen to observe the phase changes as a function of annealing time.

In this context, we report on the results of a thorough study of the magnetic and structural properties of high aspect ratio iron oxide nanorods with ferrimagnetic Fe_3_O_4_ and antiferromagnetic α-Fe_2_O_3_ phase tunability. A comprehensive understanding of the phase changes in these iron oxide nanorods will allow for the precise tailoring of these structures, enhancing the phase-specific magnetic and structural properties and giving greater control over the phase tunability of biphase iron oxide nanorods paving the way for a wider range of potential applications.

## 2. Materials and Methods

The iron oxide nanorods (NR) were synthesized by closely following the method proposed by Sun et al. [32]. The initial solutions containing 0.4 g of Hexadecylamine (HDA), 4 mL of Oleic acid (OA), and 16 mL of 1-Octanol (Sigma-Aldrich, St. Louis, MO, USA) were continuously stirred for 30 min at 55 °C. The ratio of HDA to OA which determines the aspect ratio of the NR was fine-tuned by varying the amount of HDA, based on the work of Raja et al. [25]. The resulting clear solution was then left to reach room temperature, into which 4 mL of Iron (0) Pentacarbonyl (Sigma-Aldrich, St. Louis, MO, USA) was added and stirred for another hour. The solution in a Teflon-lined container was then encased in a steel metal jacket and placed inside a 200 °C pre-heated oven for 6 h to undergo autonomous pressure resulting in elongated nanostructures. After the solution reached room temperature, the dark-colored nanostructures were retained in the solution while the yellowish supernatant was poured out. The remainder of the solution was then cleaned using ethanol and a small amount of hexane by sonicating and centrifuging and repeated at least two times or until the solute is well-separated. The well-separated product is then left to dry for a minimum of three days to obtain a fine powder. The powder is then placed in a ceramic combustion boat and inserted into a tube furnace heated to 250 °C with a continuous flow of oxygen. The samples were kept at this temperature for durations of 1, 3, 5, 7, and 9 h, and finally, all the samples were examined for their structural and compositional consistency by using the FEI Morgagni 268 Transmission Electron Microscope (TEM) (FEI, Hillsboro, OR, USA) and Bruker AXS D8 X-Ray Diffractometer (XRD) (Bruker, Madison, WI, USA), respectively. All the magnetic measurements were completed by using the Vibrating Sample Magnetometer (VSM) option in a DynaCool Physical Property Measurement System (PPMS) manufactured by Quantum Design, San Diego, CA, USA.

## 3. Results

**Figure 1:**
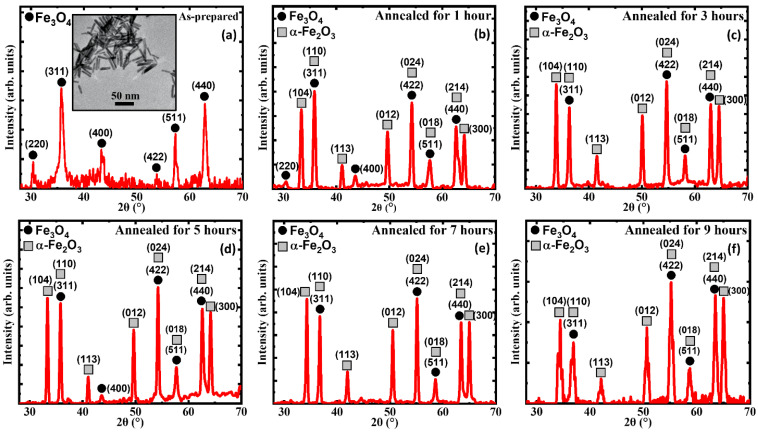
XRD patterns of the (**a**) as-prepared iron oxide nanorods (inset shows a TEM image of the as-prepared iron oxide nanorods), (**b**) 1 h annealed, (**c**) 3 h annealed, (**d**) 5 h annealed, (**e**) 7 h annealed, and (**f**) 9 h annealed. For comparison, the XRD data of (**a**) were taken from Ref. [33].

Figure 1 shows how the iron oxide nanorods changed their structural composition with annealing and additionally the inset in Figure 1a shows the TEM image of a high-quality as-prepared sample (AP) with low agglomeration, uniform shape with a size distribution of 35 nm and an aspect ratio of ~6.

A closer look at the TEM image shows that the structures are less sharp with lower contrast, which hints that the AP has not reached its full crystallinity, which is further confirmed via the XRD data. The AP which was annealed for durations of 1, 3, 5, 7, and 9 h at 250 °C shows the presence of the additional iron oxide phase of the α-Fe_2_O_3_ phase, which was not visible due to the capping provided by the Oleic Acid. The sharpness of the XRD peaks in the annealed samples (AN) is comparatively higher than the AP, which hints at the increased crystallinity with annealing [34]. Furthermore, the AN showed the consistent appearance of the biphase in the AP with annealing.

**Figure 2:**
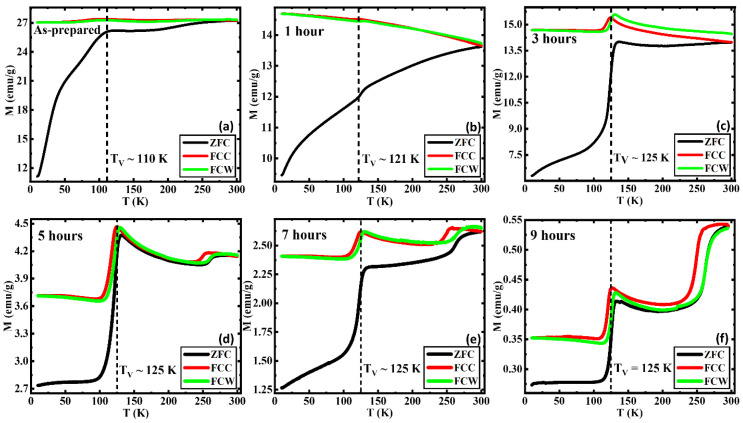
ZFC and FC M(T) curves in an applied field of 0.05 T for (**a**) as-prepared, (**b**) annealed for 1 h, (**c**) annealed for 3 h, (**d**) annealed for 5 h, (**e**) annealed for 7 h, and (**f**) annealed for 9 h iron oxide nanorods. For comparison, the M(T) data of (**a**) were taken from Ref. [33].

The temperature-dependent magnetization M(T) curves were measured following the zero-field cooled (ZFC), field-cooled (FC), and field-cooled-warming (FCW) protocols in the presence of a static magnetic field of 0.05 T. Figure 2a, which depicts a significant slope change below ~50 K, is understood to be a common phenomenon in many of the systems, specifically in ferrite nanoparticle systems [35,36,37]. The prominent magnetic shoulder found below ~50 K was confirmed via AC magnetic susceptibility measurements by Bhowmik et al. which showed that such features are due to strong magnetic spin interactions and defects in the lattice structure further enhancing the randomness in the surface spins [21,38,39,40,41]. The bifurcation between the ZFC and FC curves known as the blocking temperature is observed only in the AP below 300 K since the elevated AFM phase has increased the blocking temperature past room temperature [42,43]. The Verwey transition (VT) at which the high-temperature cubic spinel transforms into a low-temperature monoclinic structure, is a significant transition of Fe_3_O_4_, observed in all the samples between 110 K and 125 K, which can generally lie in the range of 80–125 K [44,45]. The change in the crystallographic structure is accompanied by electrical resistivity, heat capacity, magnetic susceptibility, remanence, and coercivity (H_C_) changes around the Verwey transition temperature (T_V_) [44,45,46,47]. The T_V_ tends to increase with crystallinity, plateauing at approximately 125 K. This first-order metal-insulator transition reached its peak after annealing it for 3 h at 250 °C. This implies that the samples require at least 3 h of exposure to a continuous flow of oxygen at 250 °C to reach their full crystallinity [44,48]. Furthermore, the sharp, first-order transition in samples 3, 5, 7, and 9 indicates that the Fe_3_O_4_ phase is of sufficient purity and/or stoichiometry. This is indicative of the increasing crystallinity of the Fe_3_O_4_ phase with the annealing time, and vice versa the multistage transitions below 125 K which are less sharp compared to the prior. This can be understood to be due to less crystallinity/stoichiometry in the Fe_3_O_4_ phase in the AP and 1 h AN [48]. Additionally, the suppression of the VT can occur due to slight oxidation of the Fe_3_O_4_ phase into a γ-Fe_2_O_3_-like phase which has been observed in capped nanoparticle systems [49]. The Morin transition (MT), which is a hallmark transition associated with the α-Fe_2_O_3_ (hematite) phase, can be observed in almost all the samples other than the 1 and 3 h AN. The MT is commonly known as the temperature-driven spin-flop transition and is ideally associated with a first-order magnetic phase transition in α-Fe_2_O_3_ where it transforms from a weak ferromagnetic (FM) phase with spins aligned perpendicular to the c-axis above the MT to a fully AFM phase with spins aligned parallel to the c-axis below MT along with the change of sign of the magnetic anisotropy constant [50,51,52,53,54,55]. The absence of the MT in the 1 and 3 h AN can be due to several reasons. The MT which occurs due to a subtle change of the orientation of spins in α-Fe_2_O_3_ can get affected due to impurities, defects, spin frustrations, etc. in the structure and can lead to the diminishing or disappearance of the MT [56]. The concurrent occurrence of the Morin and Verwey transitions, respectively, in samples other than 1 and 3 h annealed samples indicates the coexistence of the α-Fe_2_O_3_ and Fe_3_O_4_ phases in these samples, which was also confirmed by the XRD analysis. The α-Fe_2_O_3_ volume fraction has drastically increased in the samples that are annealed for a longer duration, as identified by the enhanced sharpness of the respective MTs. Prominent thermomagnetic hysteresis has been observed between the field-cooled cooling (FCC) and field-cooled warming (FCW) M(T) for the 5, 7, and 9 h AN and it becomes stronger in the 9 h AN compared to the other two, which also complements our hypothesis about the enhanced volume fraction of the α-Fe_2_O_3_ phase with increasing annealing duration. The significant separation between the FCC and FCW in Figure 2c indicates that the sample was able to achieve a higher magnetization at the end of FCW compared to the magnetization achieved at the start of FCC. With the spins aligned at the start of the FCC protocol, the magnetization further increases as the temperature drops since the magnetic moments align with the external field. While heating up when following the FCW protocol, the magnetic moments which did not possess enough energy to align, further aligned with the applied field leading to a separation between the FCC and FCW magnetization curves.

**Figure 3:**
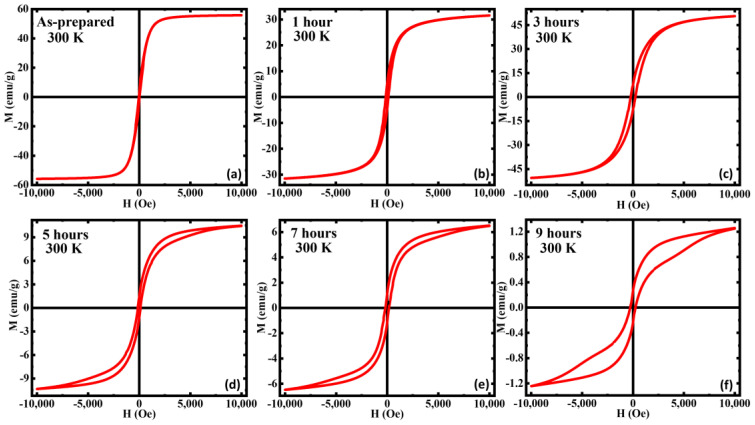
Magnetic hysteresis loops M(H) of (**a**) as-prepared, (**b**) annealed for 1 h, (**c**) annealed for 3 h, (**d**) annealed for 5 h, (**e**) annealed for 7 h, and (**f**) annealed for 9 h iron oxide nanorods at 300 K. For comparison, the M(H) data of (**a**) were taken from Ref. [33].

In Figure 3, we show the magnetization vs. the applied field M(H) at room temperature for all the samples. It is noteworthy that the magnetization at the highest magnetic field 1 T (M_S_), has dropped with annealing in all the samples except for the 3 h AN, which shows an enhancement in its magnetization. This enhancement is understood to be due to an optimization of phase coexistence and crystallinity. The AP showed the highest M_S_ along with the lowest coercivity H_C_ and room temperature superparamagnetic behavior, indicating the sample acts as a single domain at room temperature [57]. Furthermore, the AP showed a decent approach to saturation, while the others did not show full saturation even at higher fields, indicating the increased volume fraction of the AFM α-Fe_2_O_3_ phase since to achieve the saturation magnetization of FiM/AFM systems, the applied field needs to surpass the spin-flop transition, which is 10–100 T in magnitude [33,58,59]. 

This increase in the volume fraction of the AFM phase can be further observed with the decrease in the M_S_ compared to the AP. The H_C_ has increased with annealing and 3 h annealed shows the highest. The increase in H_C_ can be attributed to the strong competition between the AFM α-Fe_2_O_3_ phase and the magnetically softer FiM Fe_3_O_4_ phase in the annealed samples. Furthermore, the decrease in magnetization values can also be associated with the enhanced volume fraction of the AFM α-Fe_2_O_3_ phase. Most interestingly, for the 5, 7, and 9 h AN, the increasing branch of the isothermal M(H) loops exhibit a slope change of 5 kOe, and this behavior is more robust in the 9 h AN. The magnetization of the increasing branch of the 9 h AN shows a sudden but smooth jump above the slope change which indicates the occurrence of field-induced metamagnetic transition in this sample [60]. We believe that the appearance of this field-induced metamagnetic transition is associated with the presence of the AFM α-Fe_2_O_3_ phase. Field-induced metamagnetic transition usually occurs when an FM/FiM phase exists with an AFM phase and the occurrence of this phenomenon in the annealed samples (for durations above 5 h) can be due to the superficial spin disorder with the mixed magnetic phases and/or core-shell-like structure [61]. With the application of a magnetic field, the AFM phase reorients with FiM order giving rise to the metamagnetic transition [62]. 

**Figure 4:**
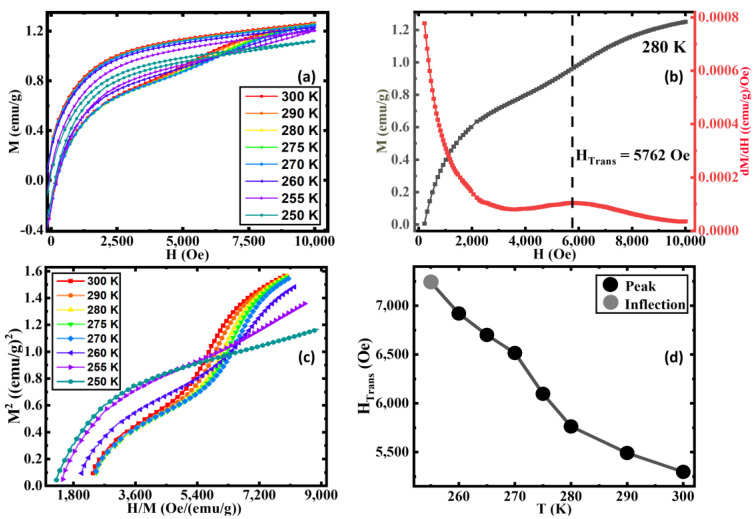
(**a**) Magnetic hysteresis loops M(H) between 250 K and 300 K, (**b**) magnetization curve and the change in magnetization with respect to field vs. field curve at 280 K, (**c**) Arrott plots between 250 K and 300 K, and (**d**) the transition field (H_Trans_) vs. temperature of the 9 h annealed sample.

For a clearer understanding, we performed M(H) measurements on the 9 h AN sample at selected temperatures above and below the MT. As shown in Figure 4a, the slope change in M(H) associated with the metamagnetic transition becomes stronger with decreasing temperatures up to ~275 K, below which it slowly weakens and disappears at T = 250 K. Usually, metamagnetic transitions are first-order transitions. To understand the order of the field-induced metamagnetic transitions in our AN sample, we show Arrott plots for 9 h annealed samples at selected temperatures in the range of 250 K ≤ T ≤ 300 K in Figure 4c. In the case of a first-order metamagnetic transition, the fourth-order coefficient of the Landau free-energy expansion becomes negative giving rise to the appearance of an inflection point/negative slope accompanied by an S-shaped curve of the Arrott plot [63,64]. It is evident that the Arrott plots for our 9 h AN exhibit S-shaped curves along with an inflection point and the negative slope in the field range of 5400 Oe ≤ H ≤ 7200 Oe at temperatures in the range of 260 K ≤ T ≤ 300 K which further confirms the occurrence of field-induced first-order metamagnetic transition in this sample [60]. The S-shape behavior of the Arrott plots disappears for T ≤ 255 K. We estimated the transition field associated with the metamagnetic transition (H_Trans_) from the first derivative of the increasing branch of the isothermal magnetization curve, as indicated in Figure 4b. Clearly, H_Trans_ shifts to higher fields with decreasing temperature and eventually disappears below 255 K, as shown in Figure 4d.

**Figure 5:**
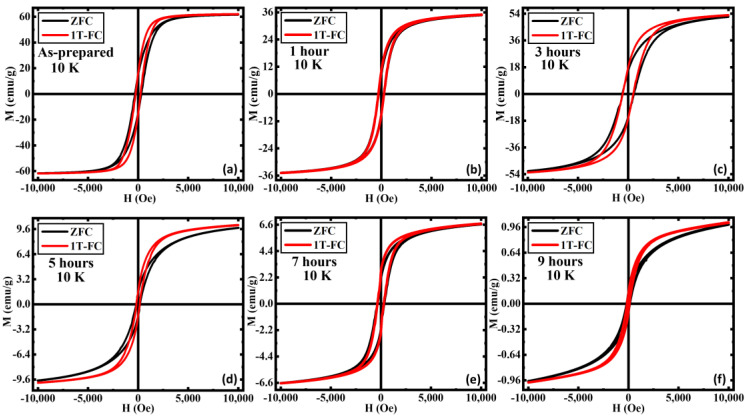
Magnetic hysteresis loops M(H) recorded with ZFC and FC protocols in an applied field of 1 T for (**a**) as-prepared, (**b**) annealed for 1 h, (**c**) annealed for 3 h, (**d**) annealed for 5 h, (**e**) annealed for 7 h, and (**f**) annealed for 9 h iron oxide nanorods at 10 K. For comparison, the M(H) data of (**a**) were taken from Ref. [33].

In Figure 5, we show the isothermal M(H) loops for all the samples at T = 10 K measured under zero-field cooled (ZFC) and field-cooled (FC) protocols with the cooling field of μ_0_H = 1 T. Under ZFC protocol, the sample was first cooled down to 10 K from 300 K in zero field, and the M(H) data were then taken at 10 K. On the other hand, under FC protocol a 1 T field was applied to the sample while cooling it down from 300 K to 10 K, and the M(H) loop was measured at 10 K. It is evident that the magnetization value at the highest applied magnetic field (1 T) remains the same for ZFC and FC M(H) loops for all the samples. Interestingly, the FC M(H) shows an enhancement magnetization in the low field region (below ~5 kOe) for all the samples in comparison to the ZFC M(H) except for the 1 h AN sample for which the aforementioned low field enhancement is comparatively smaller than the other samples. Furthermore, the enhancement in the low field magnetization in the FC M(H) loops becomes more robust as the annealing duration increases. However, we have not observed any horizontal/vertical shifts and/or increase in H_C_ in the FC M(H) loops relative to the ZFC M(H) loops for any of these samples indicating the absence of exchange bias (EB) effect in these samples.

Such low field enhancement in magnetization and the absence of exchange bias effect in these samples can be interpreted in terms of the presence of weakly ordered AFM α-Fe_2_O_3_ phase [33]. In our biphase iron oxide system, the ferrimagnetically ordered Fe_3_O_4_ phase is the dominating phase of which the volume fraction is getting replaced by the AFM α-Fe_2_O_3_ phase with increasing annealing duration. However, the sharpness of the Verwey transition strongly indicates the existence of the highly crystalline Fe_3_O_4_ phase along with the AFM α-Fe_2_O_3_ phase even in the 9 h annealed sample. At low temperatures, the total magnetic moment associated with the Fe_3_O_4_ phase contributes significantly towards total magnetization, even if the volume fraction of the aforementioned phase is small compared to the AFM counterpart of the system. Therefore, the AFM interaction among the spins associated with the AFM α-Fe_2_O_3_ phase is likely to be destabilized by the exchange field of the FiM Fe_3_O_4_ phase and hence weakens the AFM ordering of the α-Fe_2_O_3_ phase [33]. Usually, a large magnetic field (much above the spin-flop transition field of the AFM, which is typically in the range of 10–100 T) is needed in order to fully saturate a classical AFM material. However, it has been recently shown that the spin-flop transition field for the α-Fe_2_O_3_ thin films is much lower (~3 kOe) than conventional classical AFMs [59], indicating that the macro-spins associated with the AFM α-Fe_2_O_3_ can be easily saturated by applying 1 T magnetic field. In this scenario, the spins associated with the weakly ordered AFM α-Fe_2_O_3_ phase remain unaligned in the low field region when the sample is cooled in a zero-applied magnetic field and leads to a lower value of magnetization [33]. However, the spins associated with the weakly ordered AFM α-Fe_2_O_3_ phase are aligned along the field direction when the sample is cooled in a higher magnetic field (1 T) and give rise to the enhanced magnetization in the low field region.

The absence of an EB effect was also reported in a bilayer film consisting of FM Fe and metamagnetic FeRh layers grown on a MgO substrate [65]. While the Fe/FeRh/Al_2_O_3_ film showed a considerable EB due to exchange coupling between the Fe magnetic moments and the ferromagnetically arranged uncompensated sublattice magnetization of AFM FeRh at the Fe/FeRh interface, the Fe/FeRh/MgO film did not show EB because of the compensated AFM sublattice magnetization [65]. A controllable EB effect was also observed in an ion-implanted single-layer AFM FeRh film introducing a surface FM layer on top of the bulk AFM layer [66]. However, the origin of the absence of the EB effect and the existence of the field-induced metamagnetic transition in our iron oxide system with competing magnetic phases are rather different from the aforementioned case. Usually, the metamagnetic transition requires very strong magnetic fields to transform an antiferromagnetically/ferrimagnetically ordered state into a ferromagnetically ordered state. Because of the presence of the weakly ordered AFM α-Fe_2_O_3_ phase specifically near the MT, the metamagnetic transition occurs at a much lower magnetic field (also known as the spin-flop transition field of α-Fe_2_O_3_ [59]) in our Fe_3_O_4_/α-Fe_2_O_3_ system, unlike classical AFM materials. However, the strength of the AFM interaction increases gradually at low temperatures, which requires higher magnetic fields to drive the metamagnetic transition. This explains the disappearance of the metamagnetic transition in the measured field range (<1 T) below 250 K (Figure 4a).

**Figure 6:**
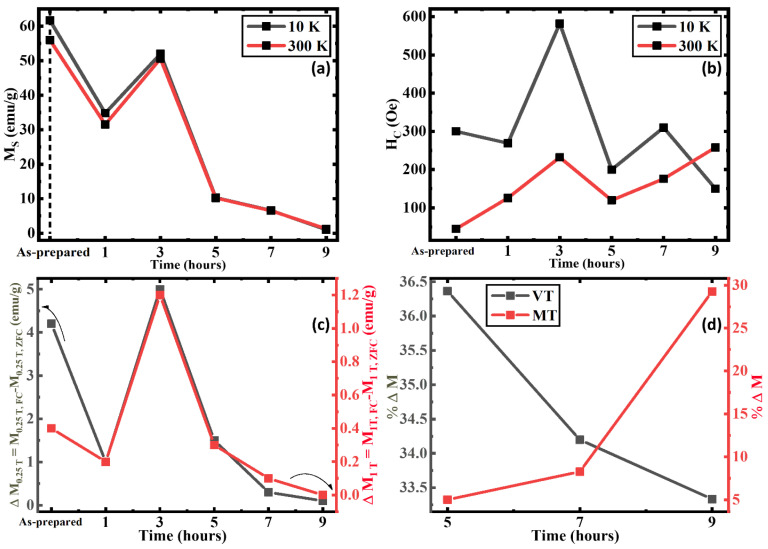
(**a**) Maximum magnetization at 1 T, (**b**) Coercivity at 10 K and 300 K, (**c**) Difference between the FC and ZFC magnetization at 10 K on 0.25 T and 1 T applied field of iron oxide nanorods, and (**d**) Percentage change in magnetization around VT and MT of 5, 7, and 9 h annealed iron oxide nanorod samples.

As shown in Figure 6a, the M_S_ value (magnetization at the highest magnetic field 1 T) at 10 K and at 300 K show variation between their respective low and high-temperature values which can be observed in the AP, 1 h, and 3 h AN. The M_S_ tends to be higher at a low temperature in comparison to a high temperature as the magnetic moments can be easily aligned with fewer thermal fluctuations [67,68,69]. Clearly, the M_S_ value for the 9 h AN sample is minimum amongst all the other samples at both 10 K and 300 K. Moreover, the difference in the M_S_ values between 10 K and 300 K significantly decreases with an increase in the annealing duration. All these observations strongly indicate an enhancement in the volume fraction of the AFM α-Fe_2_O_3_ phase with increasing durations. This can be compared with Figure 6b where the H_C_ at low and high temperatures followed the same trend until the 9 h AN where the weakly FM α-Fe_2_O_3_ phase above the MT results in a higher H_C_ at the high temperature. The enhancement of the H_C_ until the 3 h AN in both temperatures is understood to be due to the interfacial interaction between the AFM and FiM phases with the optimization of the two phases, as the spin distribution and the arrangement change [70]. Specifically, the significant enhancement of the crystallinity in the FiM Fe_3_O_4_ phase of the 3 h AN results in a better ordering of spins, which has a higher potential for influencing the spins at the interfaces as they prevent the spins from getting freely ordered with the applied field leading to the enhanced H_C_ [71]. When the AFM phase further increases, the H_C_ decreases as the interfacial interactions become directly affected by the effective area and microstructural changes between the two phases as the local spin frustrations result [70,72]. Figure 6c was calculated using

ΔM_X_ = M_X,FC_ − M_X,ZFC_ (1)

where ΔM_X_ denotes the difference in magnetization at the “X” applied field. Here, the relation highlights the difference between the FC and ZFC magnetization at 0.25 T and 1 T applied field points which clearly shows that at around 0.25 T field, the difference between the FC and ZFC magnetizations is higher, indicating the pinning effect [19]. Though the trend is similar at both the said applied fields of 0.25 T and 1 T, the AP sample is an exception as it shows a higher change in M_S_ values at 0.25 T which can be understood to be due to the significantly higher FiM Fe_3_O_4_ with the low AFM α-Fe_2_O_3_ phase. This is the reason we observe a larger difference between the magnetization in the FC and ZFC protocols in the low-field field (0.25 T) as the α-Fe_2_O_3_ phase aligns [33]. The percent change in magnetization (% ΔM) at VT and MT has been calculated by using the following equation:

% ΔM = (M_max_ − M_min_)/M_max_ (2)

We consider the significant step change of the ZFC curve at ~VT and ~MT selected by the higher and lower inflection points in Figure 2d–f. Figure 6d shows a decrease in the percentage change in magnetization at the VT while simultaneously it increases at the MT as the annealing duration increase. This implies that the volume fraction of the AFM α-Fe_2_O_3_ phase increases with the annealing duration. The percentage change in the magnetization at ~VT, though significant, is quite low as the FiM Fe_3_O_4_ phase has attained its peak crystallization at 3 h of annealing, and the decrease solely corresponds to the decrease of the FiM Fe_3_O_4_ volume fraction.

**Figure 7:**
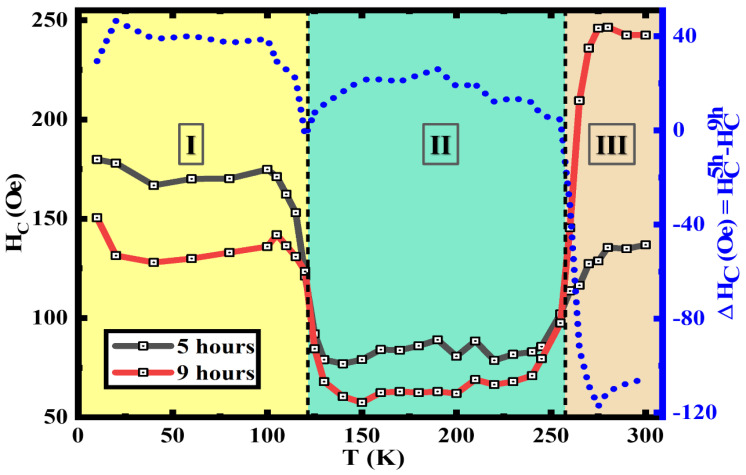
Temperature dependence of coercivity and coercivity difference between the 5 h annealed and 9 h annealed iron oxide nanorods.

Here, the temperature dependence of H_C_ of the 5 h annealed and the 9 h annealed samples are extracted from M(H) loops at varying temperatures, these clearly show the VT and the MT at ~120 K and ~260 K, respectively. As the temperature is increased from 10 K the H_C_ initially drops from a high value at around ~50 K as this has been observed to be the spin-freezing temperature in many of the nanoparticle systems [19,20,35,73]. The increased H_C_ below the VT in region I, is understood to be due to the large magneto-crystalline anisotropy change which occurs at the VT. Here, the anisotropy decreases with the temperature increment across VT, as the crystalline structure changes from monoclinic to cubic spinel, which can be observed in both samples [74,75,76]. The higher change in H_C_ was observed in the 5 h annealed sample because when the volume fraction of Fe_3_O_4_ is higher, it leads to a larger change in magneto-crystalline anisotropy across regions I and II. The biphase nanostructures between regions I to II show decreased H_C_ as the anisotropy drops above VT in Fe_3_O_4_. The increase in H_C_ at the MT when approaching region III indicates the anisotropy change associated with the MT. Here, the 9 h annealed sample shows a much higher H_C_ change across MT as it possesses structures with a higher volume fraction of α-Fe_2_O_3_ which transitioned from AFM to weakly FM when moving across MT. This phenomenon is related to the anisotropy change which is resulted from the change in magnetic moments aligning along the c-axis to perpendicular to it [19,51,77]. The difference between the H_C_ values depicted by the right-hand axis in Figure 7 clearly shows how the H_C_ of the 9 h annealed samples dominates after passing the MT in region III.

Finally, it is worth mentioning that while the magnetic properties discussed above are for the biphase iron oxide nanorods, other shapes of nanostructures or thin films with biphase correlations could also share some similar features. To verify this, future studies are needed, which is beyond the scope of the present manuscript.

## 4. Conclusions

A systematic study of the annealing-induced phase evolution of iron oxide nanorods was conducted at 250 °C for varying durations of time, enabling a closer look at how the crystallinity and the volume fractions of Fe_3_O_4_ and α-Fe_2_O_3_ can be tuned between major and minor phases. The pinning effect was observed in all the samples except the 1 h annealed sample which is understood to be due to the γ-Fe_2_O_3_-like phase inhibiting the pinning effect under the low-temperature field-cooled protocol. The disappearance of the Morin transition despite the XRD results was understood to be due to strong magnetic interactions along with impurities/defects. The annealing conducted at 250 °C for 3 h showed enhanced magnetism and pinning effects. Furthermore, the metamagnetic transition was understood to be due to the overwhelming α-Fe_2_O_3_ phase observed in 5, 7, and 9 h annealed samples, while it showed significance only in the latter. This study underscores a simple and cost-effective means for controlling the structural and magnetic properties of iron oxide nanostructures by relative phase control.

## Data Availability

The research data will be available upon request.

## References

[1] Feynman R.P. (1961). There’s Plenty of Room at the Bottom. Eng. Sci..

[2] Thiesen B., Jordan A. (2008). Clinical applications of magnetic nanoparticles for hyperthermia. Int. J. Hyperth..

[3] Kumar C.S.S.R., Mohammad F. (2011). Magnetic Nanomaterials for Hyperthermia-based Therapy and Controlled Drug Delivery. Adv. Drug Deliv. Rev..

[4] Lavorato G.C., Das R., Xing Y., Robles J., Litterst F.J., Baggio-Saitovitch E., Phan M.-H., Srikanth H. (2020). Origin and Shell-Driven Optimization of the Heating Power in Core/Shell Bimagnetic Nanoparticles. ACS Appl. Nano Mater..

[5] Gandia D., Gandarias L., Rodrigo I., Robles-García J., Das R., Garaio E., García J.Á., Phan M.-H., Srikanth H., Orue I. (2019). Unlocking the Potential of Magnetotactic Bacteria as Magnetic Hyperthermia Agents. Small.

[6] Denmark D.J., Hyde R.H., Gladney C., Phan M.-H., Bisht K.S., Srikanth H., Mukherjee P., Witanachchi S. (2017). Photopolymerization-based synthesis of iron oxide nanoparticle embedded PNIPAM nanogels for biomedical applications. Drug Deliv..

[7] Lavorato G.C., Das R., Masa J.A., Phan M.H., Srikanth H. (2021). Hybrid magnetic nanoparticles as efficient nanoheaters in biomedical applications. Nanoscale Adv..

[8] Wang C., Meyer J., Teichert N., Auge A., Rausch E., Balke B., Hütten A., Fecher G.H., Felser C. (2014). Heusler nanoparticles for spintronics and ferromagnetic shape memory alloys. J. Vac. Sci. Technol. B.

[9] Karmakar S., Kumar S., Rinaldi R., Maruccio G. (2011). Nano-electronics and spintronics with nanoparticles. J. Phys. Conf. Ser..

[10] Hvolbæk B., Janssens T.V.W., Clausen B.S., Falsig H., Christensen C.H., Nørskov J.K. (2007). Catalytic activity of Au nanoparticles. Nano Today.

[11] Raimondi F., Scherer G.G., Kötz R., Wokaun A. (2005). Nanoparticles in Energy Technology: Examples from Electrochemistry and Catalysis. Angew. Chem. Int. Ed..

[12] Chandra S., Das R., Kalappattil V., Eggers T., Harnagea C., Nechache R., Phan M.-H., Rosei F., Srikanth H. (2017). Epitaxial magnetite nanorods with enhanced room temperature magnetic anisotropy. Nanoscale.

[13] Geng R., Luong H.M., Pham M.T., Das R., Repa K.S., Robles-Garcia J., Duong T.A., Pham H.T., Au T.H., Lai N.D. (2019). Magnetically tunable organic semiconductors with superparamagnetic nanoparticles. Mater. Horiz..

[14] Au T.H., Trinh D.T., Tong Q.C., Do D.B., Nguyen D.P., Phan M.-H., Lai N.D. (2017). Direct Laser Writing of Magneto-Photonic Sub-Microstructures for Prospective Applications in Biomedical Engineering. Nanomaterials.

[15] Sangaiya P., Jayaprakash R. (2018). A Review on Iron Oxide Nanoparticles and Their Biomedical Applications. J. Supercond. Nov. Magn..

[16] Amstad E., Textor M., Reimhult E. (2011). Stabilization and functionalization of iron oxide nanoparticles for biomedical applications. Nanoscale.

[17] Manescu V., Paltanea G., Antoniac I., Vasilescu M. (2021). Magnetic Nanoparticles Used in Oncology. Materials.

[18] Wu W., He Q., Jiang C. (2008). Magnetic Iron Oxide Nanoparticles: Synthesis and Surface Functionalization Strategies. Nanoscale Res. Lett..

[19] Attanayake S.B., Chanda A., Das R., Phan M.H., Srikanth H. (2022). Emergent magnetic properties of biphase iron oxide nanorods. AIP Adv..

[20] Attanayake S.B., Chanda A., Das R., Kapuruge N., Gutierrez H.R., Phan M.-H., Srikanth H. (2022). Emergent magnetism and exchange bias effect in iron oxide nanocubes with tunable phase and size. J. Phys. Condens. Matter.

[21] Khurshid H., Phan M.H., Mukherjee P., Srikanth H. (2014). Tuning exchange bias in Fe/γ-Fe_2_O_3_ core-shell nanoparticles: Impacts of interface and surface spins. Appl. Phys. Lett..

[22] Chanda A., DeTellem D., Pham Y.T.H., Shoup J.E., Duong A.T., Das R., Cho S., Voronine D.V., Trinh M.T., Arena D.A. (2022). Spin Seebeck Effect in Iron Oxide Thin Films: Effects of Phase Transition, Phase Coexistence, And Surface Magnetism. ACS Appl. Mater. Interfaces.

[23] Chanda A., Hung C.M., Duong A.T., Cho S., Srikanth H., Phan M.H. (2023). Magnetism and spin-dependent transport phenomena across Verwey and Morin transitions in iron oxide/Pt bilayers. J. Magn. Magn. Mater..

[24] Hung C.-M., Dang D.T.-X., Chanda A., Detellem D., Alzahrani N., Kapuruge N., Pham Y.T.H., Liu M., Zhou D., Gutierrez H.R. (2023). Enhanced Magnetism and Anomalous Hall Transport through Two-Dimensional Tungsten Disulfide Interfaces. Nanomaterials.

[25] Das R., Alonso J., Nemati Porshokouh Z., Kalappattil V., Torres D., Phan M.-H., Garaio E., García J.Á., Sanchez Llamazares J.L., Srikanth H. (2016). Tunable High Aspect Ratio Iron Oxide Nanorods for Enhanced Hyperthermia. J. Phys. Chem. C.

[26] Mohapatra J., Mitra A., Tyagi H., Bahadur D., Aslam M. (2015). Iron oxide nanorods as high-performance magnetic resonance imaging contrast agents. Nanoscale.

[27] Rebolledo A.F., Bomatí-Miguel O., Marco J.F., Tartaj P. (2008). A Facile Synthetic Route for the Preparation of Superparamagnetic Iron Oxide Nanorods and Nanorices with Tunable Surface Functionality. Adv. Mater..

[28] Nguyen T.V., Luong N.A., Nguyen V.T., Pham A.T., Le A.T., To T.L. (2021). Effect of the phase composition of iron oxide nanorods on SO_2_ gas sensing performance. Mater. Res. Bull.

[29] Liu Z., Tay S.W., Li X. (2011). Rechargeable battery using a novel iron oxide nanorods anode and a nickel hydroxide cathode in an aqueous electrolyte. Chem. Commun..

[30] Cornell R.M., Schwertmann U. The Iron Oxides. Iron Oxides..

[31] Li Z., Chanéac C., Berger G., Delaunay S., Graff A., Lefèvre G. (2019). Mechanism and kinetics of magnetite oxidation under hydrothermal conditions. RSC Adv..

[32] Sun H., Chen B., Jiao X., Jiang Z., Qin Z., Chen D. (2012). Solvothermal Synthesis of Tunable Electroactive Magnetite Nanorods by Controlling the Side Reaction. J. Phys. Chem. C.

[33] Attanayake S.B., Chanda A., Das R., Phan M.H., Srikanth H. (2023). Effects of annealing temperature on the magnetic properties of highly crystalline biphase iron oxide nanorods. AIP Adv..

[34] Chauhan A., Chauhan P. (2014). Powder XRD Technique and its Applications in Science and Technology. J. Anal. Bioanal. Tech..

[35] Shankar A., Chand M., Basheed G.A., Thakur S., Pant R.P. (2015). Low temperature FMR investigations on double surfactant water based ferrofluid. J. Magn. Magn. Mater..

[36] Ghoshani M., Sánchez E.H., Lee S.S., Singh G., Yaacoub N., Peddis D., Mozaffari M., Binns C., De Toro J.A., Normile P.S. (2021). On the detection of surface spin freezing in iron oxide nanoparticles and its long-term evolution under ambient oxidation. Nanotechnology.

[37] Muscas G., Concas G., Cannas C., Musinu A., Ardu A., Orrù F., Fiorani D., Laureti S., Rinaldi D., Piccaluga G. (2013). Magnetic properties of small magnetite nanocrystals. J. Phys. Chem. C.

[38] Bhowmik R.N., Aneeshkumar K.S. (2018). Low temperature ferromagnetic properties, magnetic field induced spin order and random spin freezing effect in Ni_1.5_Fe_1.5_O_4_ ferrite; prepared at different pH values and annealing temperatures. J. Magn. Magn. Mater..

[39] Obaidat I.M., Mohite V., Issa B., Tit N., Haik Y. (2009). Predicting a major role of surface spins in the magnetic properties of ferrite nanoparticles. Cryst. Res. Technol..

[40] Narayanaswamy V., Al-Omari I.A., Kamzin A.S., Khurshid H., Khaleel A., Issa B., Obaidat I.M. (2022). Coercivity and Exchange Bias in Ti-Doped Maghemite Nanoparticles. Magnetochemistry.

[41] Khurshid H., Lampen-Kelley P., Iglesias Ò., Alonso J., Phan M.-H., Sun C.-J., Saboungi M.-L., Srikanth H. (2015). Spin-glass-like freezing of inner and outer surface layers in hollow γ-Fe_2_O_3_ nanoparticles. Sci. Rep..

[42] IBruvera J., Zélis P.M., Calatayud M.P., Goya G.F., Sánchez F.H. (2015). Determination of the blocking temperature of magnetic nanoparticles: The good, the bad, and the ugly. J. Appl. Phys..

[43] Ji J.Y., Shih P.H., Chan T.S., Ma Y.R., Wu S.Y. (2015). Magnetic Properties of Cluster Glassy Ni/NiO Core–Shell Nanoparticles: An Investigation of Their Static and Dynamic Magnetization. Nanoscale Res. Lett..

[44] Walz F. (2002). The Verwey transition–A topical review. J. Phys. Condens. Matter.

[45] Jackson M.J., Moskowitz B. (2021). On the distribution of Verwey transition temperatures in natural magnetites. Geophys. J. Int..

[46] Verwey E.J.W. (1939). Electronic conduction of magnetite (Fe_3_O_4_) and its transition point at low temperatures. Nature.

[47] Verwey E.J.W., Haayman P.W. (1941). Electronic conductivity and transition point of magnetite (‘Fe_3_O_4_’). Physica.

[48] Walz F., Kronmüller H. (2006). Evidence for a single-stage Verwey-transition in perfect magnetite. Philos. Mag. B.

[49] Schmitz-Antoniak C., Schmitz D., Warland A., Darbandi M., Haldar S., Bhandary S., Sanyal B., Eriksson O., Wende H. (2018). Suppression of the Verwey Transition by Charge Trapping. Ann. Phys..

[50] Shimomura N., Pati S.P., Sato Y., Nozaki T., Shibata T., Mibu K., Sahashi M. (2015). Morin transition temperature in (0001)-oriented α-Fe_2_O_3_ thin film and effect of Ir doping. J. Appl. Phys..

[51] Lebrun R., Ross A., Gomonay O., Baltz V., Ebels U., Barra A.-L., Qaiumzadeh A., Brataas A., Sinova J., Kläui M. (2020). Long-distance spin-transport across the Morin phase transition up to room temperature in ultra-low damping single crystals of the antiferromagnet α-Fe_2_O_3_. Nat. Commun..

[52] Özdemir Ö., Dunlop D.J., Berquó T.S. (2008). Morin transition in hematite: Size dependence and thermal hysteresis. Geochem. Geophys. Geosystems.

[53] Mørup S., Madsen D.E., Frandsen C., Bahl C.R.H., Hansen M.F. (2007). Experimental and theoretical studies of nanoparticles of antiferromagnetic materials. J. Phys. Condens. Matter.

[54] Artman J.O., Murphy J.C., Foner S. (1965). Magnetic Anisotropy in Antiferromagnetic Corundum-Type Sesquioxides. Phys. Rev..

[55] Mitra S., Das S., Basu S., Sahu P., Mandal K. (2009). Shape- and field-dependent Morin transitions in structured α-Fe_2_O_3_. J. Magn. Magn. Mater..

[56] Jaiswal A., Das R., Adyanthaya S., Poddar P. (2011). Surface effects on morin transition, exchange bias, and enchanced spin reorientation in chemically synthesized DyFeO_3_ nanoparticles. J. Phys. Chem. C.

[57] Bean C.P., Livingston J.D. (2009). Superparamagnetism. J. Appl. Phys..

[58] Chanda A., Shoup J.E., Schulz N., Arena D.A., Srikanth H. (2021). Tunable competing magnetic anisotropies and spin reconfigurations in ferrimagnetic Fe_100-x_Gd_x_ alloy films. Phys. Rev. B.

[59] Cheng Y., Yu S., Ahmed A.S., Zhu M., Rao Y., Ghazisaeidi M., Hwang J., Yang F. (2019). Anisotropic magnetoresistance and nontrivial spin Hall magnetoresistance in Pt/α- Fe_2_O_3_ bilayers. Phys. Rev. B.

[60] Paul-Boncour V., Isnard O., Guillot M., Hoser A. (2019). Metamagnetic transitions in Y_0.5_Er_0.5_Fe_2_D_4.2_ deuteride studied by high magnetic field and neutron diffraction experiments. J. Magn. Magn. Mater..

[61] Rani S., Varma G.D. (2015). Superparamagnetism and metamagnetic transition in Fe_3_O_4_ nanoparticles synthesized via co-precipitation method at different pH. Physica. B Condens. Matter..

[62] Cai X., Shi L., Zhou S. (2014). Size-dependent structure and magnetic properties of DyMnO_3_ nanoparticles. J. Appl. Phys.

[63] Inoue J., Shimizu M. (1982). Volume dependence of the first-order transition temperature for RCo_2_ compounds. J. Phys. F Met. Phys..

[64] Banerjee B.K. (1964). On a generalised approach to first and second order magnetic transitions. Phys. Lett..

[65] Suzuki I., Hamasaki Y., Itoh M., Taniyama T. (2014). Controllable exchange bias in Fe/metamagnetic FeRh bilayers. Appl. Phys. Lett..

[66] Cress C.D., Erve O.V., Prestigiacomo J., LaGasse S.W., Glavic A., Lauter V., Bennett S.P. (2023). Domain state exchange bias in a single layer FeRh thin film formed via low energy ion implantation. J. Mater. Chem. C Mater..

[67] Bloch F. (1930). Zur Theorie des Ferromagnetismus. Zeitschrift für Physik.

[68] Nadeem K., Krenn H. (2011). Exchange bias, memory and freezing effects in NiFe_2_O_4_ nanoparticles. J. Supercond. Nov. Magn..

[69] Si P.-Z., Wang X.-Y., Ge H.-L., Qian H.-D., Park J., Yang Y., Li Y.-S., Choi C.-J. (2018). Beating Thermal Deterioration of Magnetization with Mn_4_C and Exchange Bias in Mn–C Nanoparticles. Nanomaterials.

[70] Tang Y.J., Roos B., Mewes T., Demokritov S.O., Hillebrands B., Wang Y.J. (1999). Enhanced coercivity of exchange-bias Fe/MnPd bilayers. Appl. Phys. Lett..

[71] Yuan C.L. (2010). Room-temperature coercivity of Ni/NiO Core/Shell nanoparticles fabricated by pulsed laser deposition. J. Phys. Chem. C.

[72] Choi J., Wu J., Wu Y.Z., Won C., Scholl A., Doran A., Owens T., Qiu Z.Q. (2007). Effect of atomic steps on the interfacial interaction of FeMnCo films grown on vicinal Cu(001). Phys. Rev. B Condens. Matter. Mater. Phys..

[73] Kodama R.H., Berkowitz A.E., Niff E.J.M., Foner S. (1996). Surface Spin Disorder in NiFe_2_O_4_ Nanoparticles. Phys. Rev. Lett..

[74] Bickford L.R. (1950). Ferromagnetic Resonance Absorption in Magnetite Single Crystals. Phys. Rev..

[75] Moskowitz B.M., Jackson M., Kissel C. (1998). Low-temperature magnetic behavior of titanomagnetites. Earth Planet Sci. Lett..

[76] Özdemir Ö., Dunlop D.J., Moskowitz B.M. (2002). Changes in remanence, coercivity and domain state at low temperature in magnetite. Earth Planet Sci. Lett..

[77] Shull C.G., Strauser W.A., Wollan E.O. (1951). Neutron Diffraction by Paramagnetic and Antiferromagnetic Substances. Phys. Rev..

